# Predicted Hotspot Residues Involved in Allosteric Signal Transmission in Pro-Apoptotic Peptide—Mcl1 Complexes

**DOI:** 10.3390/biom10081114

**Published:** 2020-07-28

**Authors:** Parthiban Marimuthu, Jamoliddin Razzokov, Kalaimathy Singaravelu, Annemie Bogaerts

**Affiliations:** 1 Structural Bioinformatics Laboratory (SBL), Biochemistry and Pharmacy, Faculty of Science and Engineering, Åbo Akademi University, FI-20520 Turku, Finland; 2 PLASMANT Research Group, Chemistry Department, University of Antwerp, 2610 Antwerp, Belgium; Jamoliddin.Razzokov@uantwerpen.be (J.R.); annemie.bogaerts@uantwerpen.be (A.B.); 3 Department of Future Technologies, Faculty of Science and Engineering, University of Turku, FI-20014 Turku, Finland; kalsin@utu.fi

**Keywords:** B-cell lymphoma 2, myeloid cell leukemia, binding free energy, protein interaction network, allosteric-signaling pathway

## Abstract

Mcl1 is a primary member of the Bcl–2 family—anti–apoptotic proteins (AAP)—that is overexpressed in several cancer pathologies. The apoptotic regulation is mediated through the binding of pro-apoptotic peptides (PAPs) (e.g., Bak and Bid) at the canonical hydrophobic binding groove (CBG) of Mcl1. Although all PAPs form amphipathic α-helices, their amino acid sequences vary to different degree. This sequence variation exhibits a central role in the binding partner selectivity towards different AAPs. Thus, constructing a novel peptide or small organic molecule with the ability to mimic the natural regulatory process of PAP is essential to inhibit various AAPs. Previously reported experimental binding free energies (BFEs) were utilized in the current investigation aimed to understand the mechanistic basis of different PAPs targeted to mMcl1. Molecular dynamics (MD) simulations used to estimate BFEs between mMcl1—PAP complexes using Molecular Mechanics-Generalized Born Solvent Accessible (MMGBSA) approach with multiple parameters. Predicted BFE values showed an excellent agreement with the experiment (*R*^2^ = 0.92). The van–der Waals (ΔG_vdw_) and electrostatic (ΔG_ele_) energy terms found to be the main energy components that drive heterodimerization of mMcl1—PAP complexes. Finally, the dynamic network analysis predicted the allosteric signal transmission pathway involves more favorable energy contributing residues. In total, the results obtained from the current investigation may provide valuable insights for the synthesis of a novel peptide or small organic inhibitor targeting Mcl1.

## 1. Introduction

Apoptosis—programmed cell death—is an essential biological mechanism that is regulated by the B-cell lymphoma 2 (Bcl-2) family proteins [1]. Generally, Bcl-2 proteins exhibit their apoptotic activity either via pro-apoptotic proteins or their BH3 domain peptides (PAPs) or anti-apoptotic proteins (AAPs), or both [2]. The anti-apoptotic Bcl-2 family members—such as Bcl-2, Bcl-xL, Bcl-w, Mcl1 (myeloid cell leukemia 1) and Bfl-1/A1 [3]—are characterized by pathological cell survival. Thus, AAP mediates the central role as the attractive therapeutic targets in several human diseases, such as cancer and autoimmune disorders [4]. Among the Bcl-2 family members, Mcl1 appears to be a critical survival factor in several cancer pathologies [5,6,7,8,9]. The over-expression activity of Mcl1 leads to an imbalance between PAPs and AAPs. This causes the escape of cancerous cells from the natural apoptotic process, resulting in uncontrolled cell proliferation, and also multi-drug resistance [10].

Mcl1 has a large N-terminal segment with the potential to directly influence its function [11]. Moreover, the three-dimensional structure of Mcl1 is formed by tightly packed cluster of α-helices (α1 to α8). Among these, α2–α5 and α8 helices form a unique “hydrophobic or canonical binding groove” (CBG) found at the surface of Mcl1 [12]. Although the overall topology of this CBG is highly conserved among the Bcl-2 family proteins, the binding of PAPs to this CBG is highly selective. Thus, the residues lining the CBG of Mcl1 determine the uniqueness of the binding site [13].

The pro-apoptotic Bcl-2 family members, such as Bax and Bak, as well as the BH3-only domain containing activator proteins such as Bim, Bid, and Puma, have the ability to down-regulate the anti-apoptotic activity of Mcl1 [14]. The amphipathic α-helical PAPs lined by a series of conserved residues form a hydrophobic face [15] that strictly targets to the small sub–pockets (P1 to P5) present inside the CBG of Mcl1 [16]. Moreover, this hydrophobic face of PAPs plays the central role in binding partner selectivity towards the AAPs. For instance, the BH3 domain of Bim exhibits promiscuous binding features with all the AAPs, whereas the BH3 domain of NoxaA displays selective binding to both Mcl1 and A1 [17].

Due to the non-specific binding properties of the PAPs with Mcl1, developing high-affinity inhibitors—both small molecule and peptide—that can restore apoptotic activity and inhibit cancer cell progression remains a considerable challenge [18,19,20]. Different PAPs bind to the same CBG of Mcl1, but engage different residues at the binding interface that exhibit diverse binding affinities [21]. The goal here is to understand (i) how these differences in the residues still permit binding to Mcl1, and (ii) to identify the key residues responsible for heterodimerization. 

The study conducted by Ku. B. et al. [22] presented a wide range of BFE values for different PAPs targeted to anti-apoptotic Mcl1 in mice. The current investigation aims to take advantage of the existing details due to the fact that there are no other studies reported in similar targets in human. As it is well known that the mouse is a widely used model organism in various stages of drug development processes, we strongly believe that the available experimentally determined (via isothermal calorimetric method) BFE values (K_D_ in nM) could be considered as an excellent source of information for the current investigation. These details can be used to explore the mechanistic behavior of binding that could provide a plausible explanation for the Mcl1—PAP heterodimerization in humans.

In this study, we applied a variety of state–of–the–art computational methods—the Molecular Dynamics (MD) based Molecular Mechanics-Generalized Born Solvent Accessible (MMGBSA) [23] method—to probe the molecular mechanisms of binding for mouse Mcl1—PAP (hereafter mMcl1—PAP) complexes. Furthermore, in order to gain additional insight into the binding mechanism of the mMcl1—PAP complexes, the energy contribution of each residue at the interface was also estimated by residue–decomposition analysis. Moreover, we performed dynamic network analysis to predict the optimal signal transmission pathway between mMcl1—PAP complexes. Overall, the results obtained from the current research may provide valuable information about the structural requirements for the designing of next–generation peptide inhibitors to downregulate the Mcl1 activity. Additionally, these results can be compared and validated with the behavior of human Mcl1—PAP complexes in the future.

## 2. Materials and Methods

### 2.1. Starting Structures Preparation 

The NMR structures of mMcl1 complexed with Puma (PDB ID: 2ROC) [24], NoxaA (2JM6) [21] and NoxaB (2ROD) [24] were retrieved from the Protein Data Bank (PDB) [25]. There are no experimental structures available for the rest of the mMcl1—PAP complexes. Therefore, only the peptide coordinates of Bak (PDB ID: 2VOH [26]; 1.90 Å; chain B; complexed with the anti-apoptotic protein A1), Bid (PDB ID: 2VOI [26]; 2.10 Å; chain B; complexed with protein A1), Bim (PDB ID: 1PQ1 [27]; 1.65 Å; chain B; complexed with Bcl-xL), Bax (PDB ID: 2XA0 [22]; 2.70 Å; chain C; complexed with Bcl-2) and Bmf (PDB ID: 2VOG [26]; 1.9 Å; chain B, complexed with protein A1) were extracted and superposed on the NoxaB peptide of the 2ROD structure and the complexes were generated, respectively. Due to the absence of experimental structures of Hrk and Bik peptides, they were separately modeled considering the NoxaB peptide (2ROD) [21,28] structure as the template. Furthermore, to compare the computational results with the experimental values [22] all the missing residues at the N- and C- termini of the PAPs were also modeled and added to the above-mentioned peptides. In the current study, the Modeller 9v17 [29] program was used extensively to build the homology models to generate the mMcl1—PAP complexes. Additionally, the sequence comparison between the template and the target used for modeling was obtained by EBI-Clustal Omega [30]. Subsequently, to avoid steric clashes, all the mMcl1—PAP complexes were subjected to energy minimization runs performed using Schrödinger v2018-3 suite (Schrödinger, LLC, New York, NY, 2018). Next, the structures of energy minimized mMcl1—PAP complexes were further used as a starting structure for molecular dynamics (MD) simulations. 

### 2.2. System Setup and MD Simulations

In the current investigation, ten independent systems were prepared and used for MD simulations. All the prepared systems were simulated using Amber 16 suite [31] with Amber ff03.r1 [32] force field, individually. Initially, the *tleap* program available in Ambertools 16 [31] was employed to build the model systems by adding the (i) the force field parameters; (ii) the hydrogen atoms; (iii) an appropriate number of counter-ions to neutralize the unbalanced charges; and (iv) an adequate number of transferable intramolecular potential three-point (TIP3P) water molecules [33]. Then, the system was placed in an octahedral box extended by 9 Å in every dimension from the atoms of the solute to reduce the high computational cost. Subsequently, a step-by-step equilibration was performed as follows. Initially, the system was subjected to energy minimization based on the following steps: First, all the Cα atoms were constrained with a harmonic force constant of 2 kcal/mol/Å^2^, and allowing the rest of the atoms, e.g., water molecules, counter-ions and amino acid side-chains, to move freely. 500 steps of steepest descent and 1000 steps of conjugate gradient methods were applied during the minimization. Then, the system was gradually heated from 0 to 300 K, followed by density equilibration with weak restraints on the complex for 50 ps, respectively. Next, the whole system was set free i.e., without any constraints, and treated with NPT ensemble for 500 ps, while the temperature was maintained at 300 K and the pressure at 1 atm using Langevin dynamics during the simulation. Finally, considering the fact that the system built using the homology models may require a certain time period to reach dynamic stability, a 100 ns production run was carried out separately on all ten mMcl1—PAP complexes. Furthermore, to obtain reliable BFEs comparable with experimental values for all ten mMcl1—PAP complexes, the MD simulations were extensively sampled by (i) obtaining an average snapshot from the final phase of the 100 ns trajectory and used as a starting structure, (ii) the production run was extended to 25 ns with ten repeats each, yielding a total of 3.85 µs; (iii) all subsequent analyses were performed using the trajectories obtained from the extended time period (i.e., 25 ns). During the simulations, 2 fs time step integration was implemented for the entire simulation. The SHAKE algorithm [34] was used to constrain all hydrogen atoms. The 8 Å cut-off was applied onto the short-range—electrostatic and van der Waals—interactions and monitored every step, and the particle-mesh Ewald [35,36] method was applied to monitor the long-range electrostatic interaction at every third step. Periodic boundary conditions were applied to all dimensions of the system. 

### 2.3. Binding Free Energy (BFE) Estimation for mMcl1—BH3 Peptide Complexes

The BFEs were estimated with the Molecular Mechanics-Generalized Born Surface Area (∆G_MMGBSA_) approach, which is a post-processing trajectory analysis technique that is performed using MMPBSA.py [23] program available in the Amber16 package [31]. In the current study, three different GB parameters—Tsui and Case (igb = 1 [37], Onufriev et al. (igb = 2 (α = 0.8, β = 0.0, γ = 2.909) and igb = 5 (α = 1.0, β = 0.8, γ = 4.85) [32,38,39]—were used to compute the BFE values. The α, β and γ represents rescaling parameters for the solvation effects, while the igb represents the implicit solvent model used for estimating the polar solvation free energy from the simulation [32]. This approach is aimed to provide the crucial details on the thermodynamic energy components responsible for the binding, which were shown in diverse biological systems [40,41,42,43]. Similarly, the BFEs were estimated for all mMcl1—PAP complexes using the 500 snapshots retrieved from the last 10 ns of the MD trajectory with an even interval of 20 ps. 

Here, the BFEs were estimated for all mMcl1—PAP complexes using the following equation:

ΔG_bind_ = G_complex_ − G_protein_ − G_peptide_ (1)

where ΔG_bind_ represents the BFEs of the mMcl1—PAP complex; and G_complex_, G_protein_ and G_ligand_ represent the BFEs of the mMcl1—BH3 complex, mMcl1 and PAPs, respectively. 

The energies were calculated based on the following equation:

ΔG = ΔG_gas_ + ΔG_sol_ − TΔS (2)

where ΔG_gas_ corresponds to the molecular mechanic component in the gas phase, ΔG_sol_ corresponds to solvation of the BFEs, and –TΔS corresponds to the change of conformational entropy due to PAP binding. Generally, the entropic term in the MMGBSA approach is estimated with normal mode analysis (NMA) or quasi-harmonic analysis (QHA) [44] based on their vibrational frequencies. In the current study, the estimation of the conformational entropy was ignored for the following reasons: (i) the main aim of the current investigation is to obtain the relative BFE values and not to acquire the absolute BFEs, (ii) over-estimation of entropy values by NMA approach, [45] (iii) hard to achieve low convergence values by QHA approach [46,47,48] and (iv) its high demanding computational cost [43,49]. It is also reported that the entropic component is not required for similar kind of binding partners [50,51]. 

The molecular mechanics (ΔG_gas_) components of the BFE estimation can be obtained by the following equation:

ΔG_gas_ = ΔG_ele_ + ΔG_vdW_ (3)

where ΔG_ele_ corresponds to electrostatic interaction and ΔG_vdW_ corresponds to the van der Waals interaction. The contributions of the solvation free energy for the BFE estimation can be obtained by the following equation:

ΔG_sol =_ ΔG_pol.sol_ + ΔG_nonpol.sol_ (4)

where ΔG_pol.sol_ corresponds to polar solvation term that can be obtained by MMPBSA or MMGBSA approaches, and ΔG_nonpolar_ corresponds to the nonpolar solvation term. 

Furthermore, the nonpolar component for the BFE estimation was calculated using the following equation:

∆G_non-polar_ = *γ* • SASA + *β* (5)

where, the SASA corresponds to solvent-accessible surface-area, and the *γ* and *β* are surface tension and regression off-set of the linear relationship, and the values were set to 0.0072 kcal/mol•Å^−2^ and 0.92 kcal/mol [52,53], respectively. The default dielectric constant parameters were used: 1 and 80 for the interior solute and the exterior solvent, respectively.

### 2.4. Per Residue Decomposition (PRD) Analysis for mMcl-1—PAP Complexes

To identify the hotspot residues responsible for PAPs binding to mMcl1, the PRD analysis was performed on each residue of all the mMcl1—PAP complexes using the *decomp* [23] module of the Amber 16. The binding interaction of each mMcl1—PAP complex comprises four important energy terms: van der Waals contribution (ΔG_vdW_), electrostatic contribution (ΔG_ele_), polar solvation contribution (ΔG_polar_), and non-polar solvation contribution (ΔG_nonpolar.sol_) (Equation (3)).

ΔG_inhibitor-residue_ = ΔG_vdW_ + ΔG_ele_ + ΔG_polar_ + ΔG_nonpol.sol_ (6)

where the vdW and electrostatic interactions between mMcl1 and PAPs were computed using MMPBSA.py program in Amber 16. All the energy components were calculated using the same 500 snapshots retrieved from the last 10 ns of the MD trajectory with an even interval of 20 ps used for BFE calculations.

### 2.5. Trajectory Analysis

All the trajectories obtained from the MD simulations were analyzed using the *cpptraj* [54] program available in Amber suite. The *hbond* program available in Amber tools was used to estimate the evolution of polar interactions over the time period with the distance and the angle cut-off to 3.2 Å and 120°, respectively.

### 2.6. Dynamic Network Analysis 

The dynamic network analysis aimed to provide both, inter and intra molecular contact network of a given complex, and in particular, strength of a specific interaction between the subunits, which can be obtained from the MD trajectory. To perform the dynamic network analysis, the MD trajectory corresponding to a PAP that exhibited a high binding affinity with mMcl1 (mMcl1—Bak complex; K_D_ = 1.33 (nM) (Table 1)) was alone selected as a representative. Here, the *Networkview* plugin [55] available in the VMD [56] program was implemented to explore the allosteric signal transmission pathway using the MD trajectory corresponding to the mMcl1—Bak complex as the representative. 

In the process of building a network using a protein-protein complex, every Cα atom was represented as *“nodes”*, while the pairwise contacts between these Cα atoms were represented as *“edges”* that are associated with weights based on correlated motions. These nodes and edges are the essential elements required for a reliable allosteric pathway/network construction. The *“dynamic network”* is obtained when the set of nodes and edges were connected to another node for more than 75% of the time period within 4.5 Å distance. The pairwise contacts between the Cα atoms can be calculated using the following cross-correlation coefficient equation (*X_ij_*).

(7) $$\begin{array}{ccc} X_{ij}\; & = & \frac{\langle (r_{i}-\langle r_{i}\rangle )•(r_{j}-\langle r_{j}\rangle )\rangle }{\sqrt{{\langle (r_{i}-\langle r_{i}\rangle )\rangle }^{2}{\langle (r_{j}-\langle r_{j}\rangle )\rangle }^{2}}} \end{array}$$

where, *r_i_* and *r_j_* represent the displacement of the *i*th and *j*th atoms with respect to the corresponding atoms from the averaged structure, and the angle brackets represent the averaged values. 

Additionally, the distances (*d_ij_*) are calculated using the following equation.

(8) $$\begin{array}{ccc} d_{ij} & = & -\log\left(\left|X_{ij}\right|\right) \end{array}$$

where *i* and *j* correspond to the distance between two nodes.

Subsequently, the Girvan-Newman algorithm embedded in the plugin splits the constructed network into communities, where the interconnected nodes are displayed in compact and/or scattered fashion [57]. These communities comprise a number of critical nodes (betweenness) that are associated with modularity scores. Further analysis on these critical nodes situated at the interface and the connecting edges of the neighboring communities are predicted as optimal and sub-optimal community network, i.e., the potential route for signal transmission.

## 3. Results and Discussion

**Figure 1:**
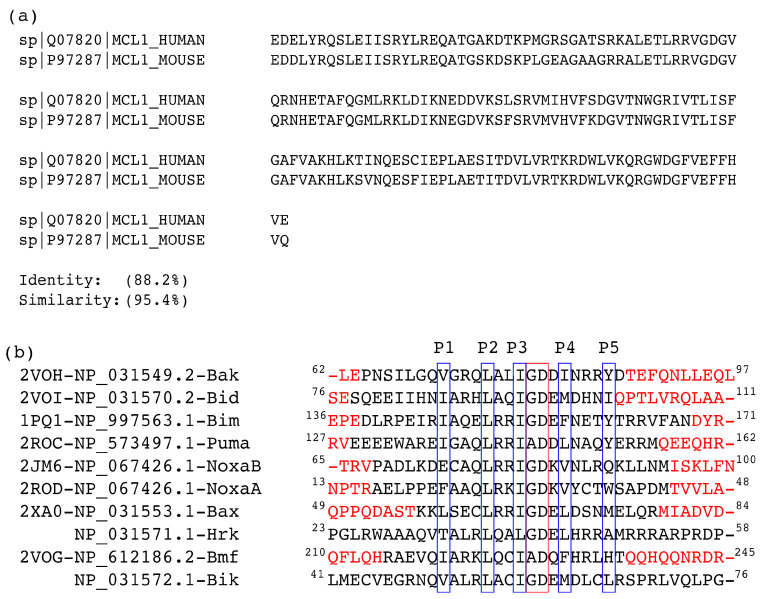
(**a**) Sequence comparison between human and mouse anti-apoptotic Mcl1 protein; (**b**) Structure-based sequence alignment of the PAPs used in this study. P1, P2, P3, P4 and P5 labels (blue boxes) indicate the five conserved hydrophobic residue positions, the highly conserved GD doublet motif (red box) and the residues highlighted in red color represents modeled regions.

A wide variety of binding affinity data is available for different PAPs targeting to Mcl1 from mice, which is absent for the human case [22]. This led us to focus on the interactions between mouse (m)Mcl1 and PAP peptides. This is a reasonable approach to gain insight into the human case, because of the high sequence identity between human and mouse Mcl1 (Figure 1a).

**Figure 2:**
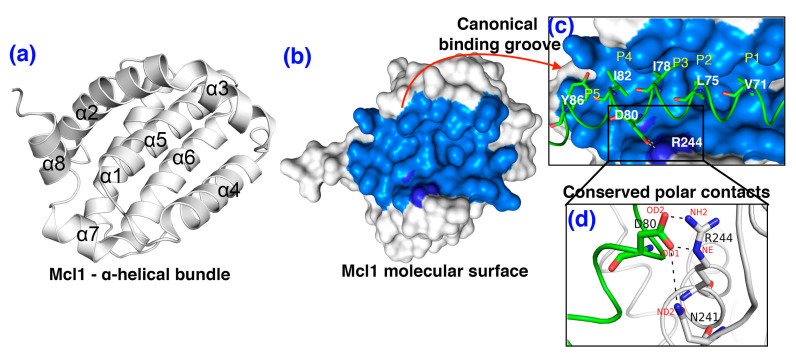
The three-dimensional model of mMcl1—Bak complex: (**a**) the cartoon representation highlighting the α-helical bundle (α1 to α8) of Mcl1. (**b**) Molecular surface representation of Mcl1 (white) highlights the canonical hydrophobic binding groove (CBG) (marine blue) where its binding partner exhibits interaction at various range of binding affinity. (**c**) The high affinity mBak peptide (K_D_ = 1.33 nM; green; cartoon loop) [22] bound to the CBG (marine blue) of Mcl1. (**d**) The magnified image of the conserved polar interaction between the residues D80 of mBak peptide and N241 and R244 of mMcl1, respectively.

Initially, the primary sequences of human and mouse Mcl1 and PAP were compared. The sequence comparison shows a high sequence identity (88.2%) between mouse and human Mcl1 (see Figure 1a) and the hydrophobic residues present at the interface region of PAPs (see Figure 1b) were highly conserved. Therefore, the selected mMcl1 and PAP sequences were used to build ten different mMcl1—PAP model complexes (see Figure 2 and Supplementary Figure S2) using the homology-modeling approach that generated 100 models for each complex. Subsequently, the interface regions of the mMcl1—PAP models were closely analyzed. The analysis shows that the highly conserved residues of the PAP models form a hydrophobic face of the amphipathic α-helices (see Figure 2c). These hydrophobic face residues promote tight binding of PAP to the binding pocket of mMcl1. Therefore, the mMcl1—PAP complexes were used as starting coordinates for the MD simulations. 

Previously, several studies have been carried out on different Bcl-2 family proteins using various MD simulation approaches. These investigations revealed (i) the driving force behind the intra-molecular conformational change [58], (ii) the helix stability [59,60], (iii) crucial residues involved in heterodimerization [61] (iv) crucial molecular properties responsible for complexity [62,63,64] (v) hot-spot residues [65] (vi) effects of Bim mutants [66] and (vii) the inter-helical interactions across families [67]. Based on this information, we attempted to explore the mMcl1—PAP complexes to extend our understanding of the molecular mechanism of heterodimerization by identifying the key features. To achieve this, an extensive sampling of MD simulations was carried out on ten different mMcl1—PAP model complexes in explicit solvent conditions. Subsequently, the MD simulation results were used to calculate the binding free energy (BFE), estimate the per-residue decomposition (PRD) and perform dynamic network analysis.

### 3.1. Conformational Stability Investigation of mMcl1—PAP Complexes during MD Simulations

**Figure 3:**
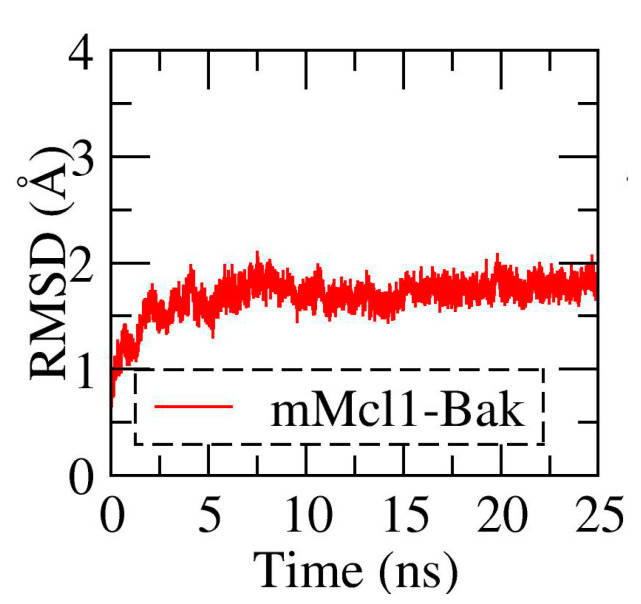
The root-mean-squared-deviation (rmsd) of mMcl1—PAP complex.

In order to examine the conformational stability of each mMcl1—PAP complex during the simulation time, the root-mean-squared deviation (rmsd) analysis was performed by extracting the Cα atoms from each snapshot and compared to its initial frame (Figure 3 and Supplementary Figure S3a). These graphs exhibits, after the short relaxation phase, the mBak, mBid, mBim, mPuma, mNoxaA, and mHrk—mMcl1 complexes gradually reached the equilibrium ~2 Å deviation. This stable equilibrium was maintained until end of the simulation. Likewise, the mNoxaB, mBax and mBik—mMcl1 complexes gradually attained the stable equilibrium state at ~3 Å deviation from its initial frame. On the other hand, the mMcl1—Bmf complex exhibit the longer time period (~10 ns) to reach the equilibration state ~2.5 Å.

Overall, from Figure 3, and Supplementary Figure S3, the rmsd values did not show any drastic fluctuation during the MD simulation and are reliable during the equilibrium phase. Therefore, the snapshots collected during the last 10 ns of the MD simulations were alone used for subsequent analysis.

### 3.2. The Polar Contacts Estimation at the mMcl1—PAP Interfaces During MD Simulations

**Figure 4:**
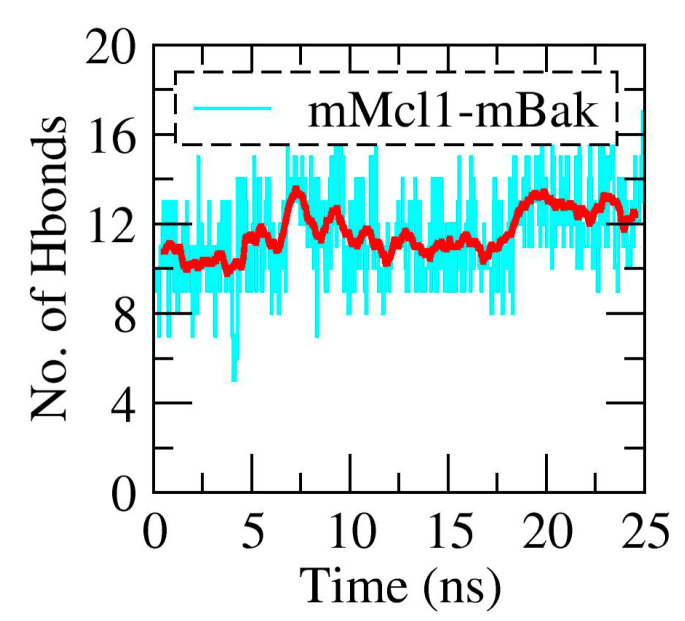
Evolution of the interdomain polar interactions (cyan) with average (red) over the period of time (ns).

Studies demonstrate that the polar contacts formed between the conserved aspartic acid residue present in the PAPs, and the arginine and asparagine residues lining the CBG of Mcl1 maintains complex stability (Figure 2d and Supplementary Figure S4) [12,21]. Additional residues were also involved, and this strengthens the complex stability, via the hydrogen bonds formation at the interface region (Supplementary Table S1). Subsequently, the total number of polar contacts at the interface region was estimated over the period of time from the MD trajectories corresponding to each mMcl1—PAP complexes (Figure 4 and Supplementary Figure S5). 

From Figure 4, it is observed that the mMcl1—Bak complex (most favorable experimental binding affinity value; K_D_ = 1.33 nM) exhibited highest number of polar contacts (~11 to 13) at the interface region during the simulation. For the peptide that exhibited relatively weak experimental values such as Puma and NoxaB (K_D_ = 2.62 and 14 nM), the total numbers of polar contacts were also found decreased (~8 to 10) in comparison with mBak peptide. On contrary, the mNoxaA, mBax, mHrk, and mBik peptides showed the low in experimental values (K_D_ = 36.9, 39.5, 44.8 and 658 nM). This effect was clearly observed in our result, i.e., the total numbers of polar contacts are significantly reduced (~lesser than 8). Overall, it is observed that the total numbers of polar contacts are higher for the complexes that have higher experimental values, while the number of polar contacts is gradually decreasing, as the experimental binding affinity values decreases. 

### 3.3. Energy Contributions Responsible for mMcl1—PAPs Heterodimerization

The study conducted by Ku. B et al. demonstrated a range of binding affinity values for various PAPs binding to mMcl1 [22]. All these binding affinity values are reported in nanomolar range. Among those the mBak and mBik peptides showed tight and weak binding affinity values targeting to mMcl1 protein, respectively, while the binding affinity value for mBad was not determined (Table 1). These experimentally determined binding affinity values provide the advantage to rank the PAPs binding to mMcl1. Due to the fact that the role of Mcl1 is significant in apoptotic regulation, it is necessary to construct novel peptides or non-peptide organic small molecule inhibitors with the potential to downregulate its activity. An efficient approach to attain this requirement is by employing advanced computational programs that can readily predict the BFE values using three-dimensional coordinates. 

There are several other methods that have been developed to calculate BFEs such as Free-Energy Perturbation (FEP) [68], Replica-Exchange Free-Energy Perturbation (RE-FEP) [69], thermodynamic integration (TI) [70], and umbrella sampling (US) [71]. These methods are computationally very expensive, they poorly converge and require extensive sampling to explore all intermediate states, while the MM(GB/PB)SA approach can estimate the BFE values relatively fast compared to the above mentioned methods, without sampling the intermediate states. Moreover, our method uses the implicit water model for calculating the free energies, which helps to avoid large fluctuation in the model system and makes it computationally efficient. In addition, our method can rigorously decompose the total BFEs into individual energy components.

The MMPBSA.py [23] program available in Amber has the advantage to predict the BFE values for the protein and its binding partners using multiple snapshots obtained from MD simulations. Here, the relative BFE values were estimated for ten different modeled mMcl1—PAP complexes that share a common CBG. The BFE values were estimated using 500 snapshots obtained from the last 10 ns of the MD simulations with an even interval of 20 ps. In order to extensively sample the conformational space for the simulated complexes, three different GB models developed by Tsui and Case (igb = 1) [37] and Onufriev et al. (igb = 2 and igb = 5) [32,38,39] were employed here. It was observed that the predicted BFE values thus achieved were negative for all studied complexes (Table 1). 

**Figure 5:**
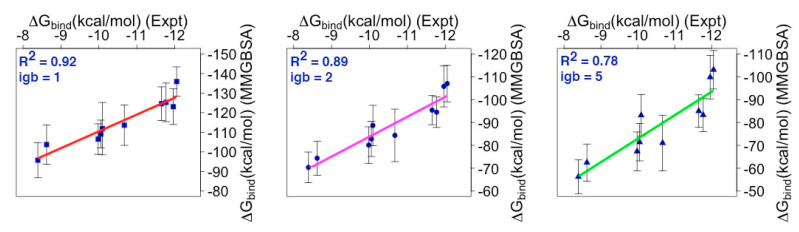
Correlation coefficient (R^2^) calculation performed using experimental [22] versus predicted BFE values. The predicted BFE values were generated using three different GB models (ref. method).

Subsequently, these predicted values obtained by the MMGBSA approach were then compared with experimental binding affinity values (Figure 5).

For igb = 1, the correlation graph (Figure 5) displays the magnitude of the predicted values in sequential increase with respect to the experimental binding affinity values. Therefore, the *R*^2^ value obtained for the igb = 1 model is as high as 0.92. In contrast, the correlation graph corresponding to igb = 2 and 5 models failed to display the sequential increase for the predicted BFE values with respect to the experimental values. Therefore, the *R*^2^ values were significantly reduced to 0.89 & 0.78 for the igb = 2 and 5 models, respectively. The reason behind this significant decrease in *R*^2^ values is because the igb = 2 and 5 models did not predict the appropriate BFE values (i.e., the energies fluctuated) for NoxaA and NoxaB—mMcl1 complexes (Table 1) with respect to the experimental values. Moreover, the igb = 5 model did not predict the appropriate BFE value for the mMcl1—Bim complex as well. Instead, all the predicted BFE values obtained using the igb = 2 and 5 models exist within the range. Overall, (i) the predicted BFE values obtained using the igb = 1 model are high, in comparison with the igb = 2 and 5 models, and (ii) the snapshots used to estimate the BFE values exhibit a plausible assumption of the binding conformation for all the mMcl1—PAPs complexes. 

Despite the fact that the MMGBSA approach accurately predicts the relative BFE values, it is also reported that the energy values obtained may not converge consistently. Therefore, it is suggested that multiple simulations are required to obtain a reliable BFE value [43]. Here, to attain a good estimate of the BFE values for the mMcl1—PAP complexes, MD simulations were carried out with ten repeats, individually. 

Collectively, the predicted BFE values obtained for the mMcl1—PAP complexes were in good agreement with the experimental values. Here, the correlation coefficient (*R*^2^) method is used to (i) quantify the consistency of the predicted BFE values, (ii) rank the PAPs with respect to the experimental binding energy values, (iii) highlight the inappropriate energies that affected the *R*^2^ values, (iv) highlight the GB model that showed higher *R*^2^ value, and (v) select the GB model that can be considered for further analysis. Note that, the eventual roles of conformational changes are not considered in our BFE calculations. In order to obtain such conformational change, the long range enhanced simulation techniques, e.g., replica exchange molecular dynamics or metadynamics may be required. These simulation techniques are computationally very expensive; therefore, we mainly focused on relative BFE estimation using the MMGBSA method. It has been noticed earlier that the length of the MD simulation is not crucial in the MMGBSA analysis, and even much shorter simulation time than we have used can provide meaningful data [72,73].

### 3.4. Energy Contributions from the Individual Components Responsible for mMcl1—PAPs Hetero-Dimerization

In the current study the igb = 1 model exhibited higher correlation value (*R*^2^ = 0.92). Therefore, the BFE values obtained using the igb = 1 model is further used to acquire the details on the individual energy components responsible for mMcl1—PAP complex formation, while the rest of the models were ignored. These details on energy contributions may be beneficial for novel peptide or non-peptide small molecule synthesis that contain the potential to specifically target Mcl1 to downregulate its activity. Subsequently, to understand the binding process of mMcl1—PAP complexes in detail, the total BFEs were fragmented into the individual energy components (Table 2). 

The result shows that the van der Waals (ΔG_vdw_), electrostatic (ΔG_ele_) and the molecular mechanics (∆G_gas_) components contributed higher favorable energies for the complex formation. Additionally, the (∆G_non-polar_) non-polar energy component also contributed favorable energy to the complex formation, but to a lower extent. In contrast, the polar (∆G_pol_) and solvation (ΔG_sol_) energy component contributed unfavorable energies to the complex formation. Closer observation on all the energies shows that the electrostatic and polar energy contribution for mBax and mBmf complexes displayed significantly low energies in comparison with the other complexes. In summary, the major favorable contributions to the total BFE of the mMcl1—PAP complexes result mainly from the van der Waals (ΔG_vdW_), electrostatic interactions (ΔG_ele_) and the molecular mechanics (∆G_gas_) components, respectively.

### 3.5. Energy Contributions from PAPs Key Residues Responsible for Heterodimerization 

**Figure 6:**
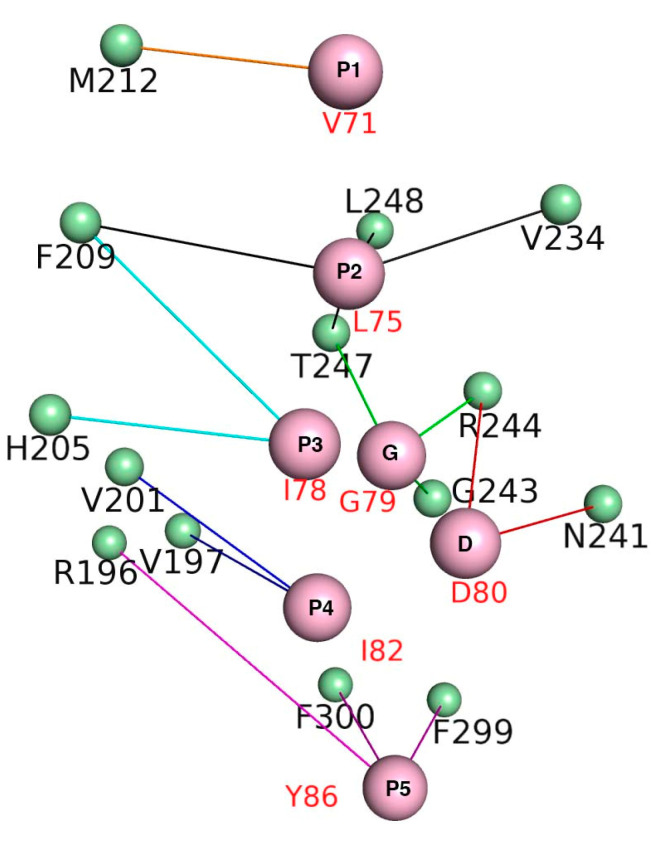
The Residue Interaction Network (RIN) shows the five conserved hydrophobic pockets (P1 to P5 and GD doublet in pink) in PAP interacting with mMcl1 (green) in three-dimensional space.

In order to design a novel peptide or non-peptide small organic molecule inhibitor with the capacity to specifically target and downregulate the Mcl1 activity, it is necessary to gain knowledge of the energy contributions of each residue, especially the residues present at the interface region of Mcl1 and its binding partners. Consequently, per-reside decomposition (PRD) analysis was performed using the *decomp* module available in Amber. Figure 6 highlights the residue interaction network (RIN) for the mMcl1—PAP complex interface projected in three-dimensional space. This RIN graph clearly shows five conserved hydrophobic pockets (P1 to P5) of PAPs and their surrounding contacts from mMcl1. Thus, the residues present in the RIN graph were used as the representative contacts for the mMcl1—PAP complexes.

**Figure 7:**
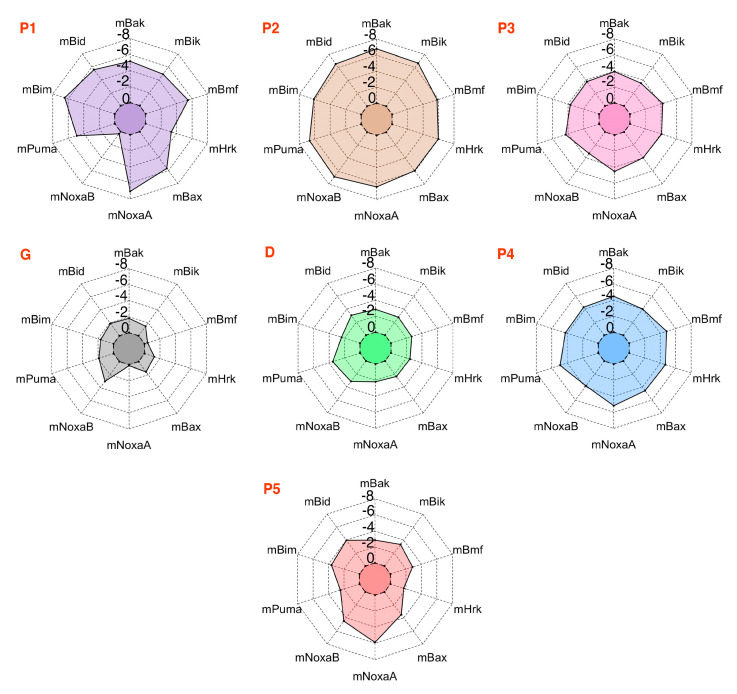
The per-residue decomposition (PRD) analysis for the conserved residues of PAPs interacting with mMcl1.

Subsequently, the BFE values were obtained for the mMcl1—PAP complex interface residues using 500 snapshots from the last 10 ns of the MD simulations with an even interval of 20 ps. The result obtained from the decomposition analysis might provide valuable insight for better understanding of the molecular basis of hetero-dimerization. Previous studies explained that the conserved residues of the amphipathic α–helical PAPs form a hydrophobic face, which has the capacity to exhibit tight binding with the CBG of Mcl1 [16]. Accordingly, the BFEs of the conserved hydrophobic residues of PAPs were extracted from the total energies (Table 3 & Figure 7). 

Among the highly conserved residues, the leucine present at the P2 position of PAPs contributed the highly favorable energies in comparison with other interface residues (Table 3). The overall energy contributions are more favorable for all PAPs complexed with mMcl1. This highly favorable energy contribution by the leucine present at the P2 position suggests that this residue plays a crucial role for the mMcl1—PAP complex formation. Additionally, the RIN graph clearly demonstrates that the leucine present at the P2 position of PAP is surrounded with a maximum of four residues—F209, V234, T247 and L248—from mMcl1. These surrounding residues play a vital role in the tight binding. Furthermore, the previous studies demonstrated that the mutation induced at this conserved leucine residue disrupts the binding significantly [7,74,75,76].

Next, the PRD analysis shows that the residues present at the P4 position of PAPs exhibited relatively less favorable impact in comparison with the residue at the P2 position. The sequence comparison for PAPs (Figure 1b) reveals that the residues present at the P4 position are all hydrophobic, but not conserved. However, the energy contributions from the PAP residues at the P4 position are more favorable when complexed with mMcl1 (Table 3). It is important to mention that, among the hydrophobic residues present at the P4 position of the PAPs, the phenylalanine and leucine residues contributed a highly favorable energy. This favorable energy contribution helps to predict that the substitution of bulky aromatic residue such as phenylalanine or aliphatic residue such as leucine at the P4 position of PAPs might be an ideal choice for the mutation to improve the tight binding with mMcl1. This prediction needs further experimental validation. Furthermore, the RIN graph displays that the residues at the P4 position established contact with V197 and V201 from mMcl1 to maintain tight binding.

The isoleucine residue present at the P3 position of PAPs displays high conservation, but the BFE values did not exhibit high energies in comparison with P2 and the P4 positions. In contrast, relatively low energies were observed at this position (Table 3). Due to inconsistency in the energy pattern, it is difficult to make any plausible assumption for the improvement of novel PAPs binding affinity. It is noted that isoleucine is replaced with leucine in P3 position of mHrk peptide that exhibited −4.08 kcal/mol. The RIN graph highlights that the residue at P3 position of PAPs interacts with two aromatic residues —H205 with imidazole side chain, and F209 with bulky indole side chain—of mMcl1. 

The sequence comparison of PAPs revealed that the P1 position did not exhibit sequence conservation. Nevertheless, the P1 position of PAPs is fairly occupied by the combination of hydrophobic and polar residues. The average BFE values obtained for the hydrophobic and polar residues are −4.94 and −1.59 kcal/mol, respectively. From this it is clearly understood that the presence of a polar residue at the P1 position severely affects the BFE value approximately by half fold, in comparison with the other hydrophobic residues. Specifically, glutamate in mNoxaB is more polar and acidic in nature over threonine in mHrk, which clearly reflected the BFE values that contributed only a smaller energy than the other. In total, the energy values obtained from the PRD analysis at P1 position clearly show that a hydrophobic residue, particularly a residue that contains a bulky aromatic side chain was highly favored over the other residue types. Moreover, the RIN graph displayed that the residues at the P1 position of PAPs mostly established the tight contact only with M212 residue from mMcl1. 

The sequence analysis at the P5 position pointed out that the residues present at this location are similar to the P1 position, i.e., the P5 position is also occupied by the combination of hydrophobic and polar residues. In addition, the aromatic residues were also observed at this position (P5) in comparison with residues present at the other positions (P1–P4). The PRD analysis performed on the residues located at the P5 position exhibited energies in huge variations, i.e., the energies range between −1.71 to −5.89 kcal/mol. In contrast, a general view on the spider plot displays the residues present in P5 position exhibited only a relatively lower impact in comparison with the residues involved at the other positions. Overall, it is difficult to make a plausible assumption due to the inconsistency in the energy pattern. Additionally, the RIN graph displayed that the residue at P5 position of PAPs interacts with the residue that contains bulky aromatic (F299 and F300) and guanidinium (R196) groups of mMcl1, respectively. 

It is reported that in addition to the conserved hydrophobic residues the GD doublet present in the P3 + 1 position of PAPs also has a significant role in the structural stability and selectivity [21]. Therefore, exploring the BFE contributions of these residues is also highly necessary. Accordingly, the BFE values of the GD doublet were extracted from the total energies. 

Similarly, aspartate present at the P3 + 2 position of PAPs has a very important role. Several studies have demonstrated that the highly conserved aspartate present at the P3 + 2 position of PAPs constantly form hydrogen bond and salt bridge interactions with carboxamide side chain of asparagine and guanidinium side chain of arginine residues [17,21]. These residues are located at the edge of the CBG of Mcl1. Accordingly, the average BFE value for the aspartate residue is approximately −2.0 kcal/mol. The aspartate residue exposed to the surface of PAPs constantly exhibited a moderate impact for the complex formation. 

Overall, the predicted BFE values obtained for all the hydrophobic residues (P1 to P5) present at the interface region clearly demonstrate that these residues in PAPs acts as the “key initiating factor” for the complex formation. Furthermore, the rest of the residues present in the peptides may provide collective support to the complex stability. 

### 3.6. Energy Contributions from mMcl1 Key Residues Responsible for Heterodimerization

**Figure 8:**
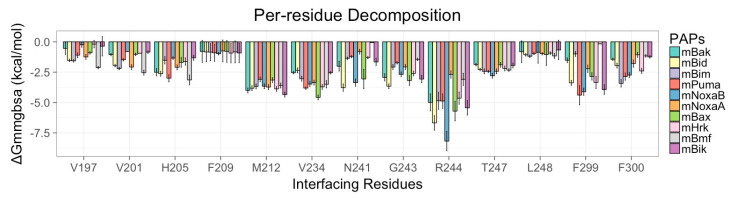
PRD analysis for mMcl1 residues interacting with PAPs.

The study conducted by Ku. B et al. [22] demonstrated that the PAPs establish tight contact at the CBG present at the surface of mMcl1 (Figure 2c and Figure 3), which plays the central role in the binding partner selectivity; however, the mechanism of selectivity remained unresolved. Therefore, exploring the BFE contributions for the residues involved in the CBG—V197, V201, H205, F209, M212, V234, N241, G243, R244, T247, L248, F299, and F300—of mMcl1 is highly necessary. Accordingly, only the BFEs for the selected residues were extracted from the total energies (Table 4) and compared (Figure 8).

**Figure 9:**
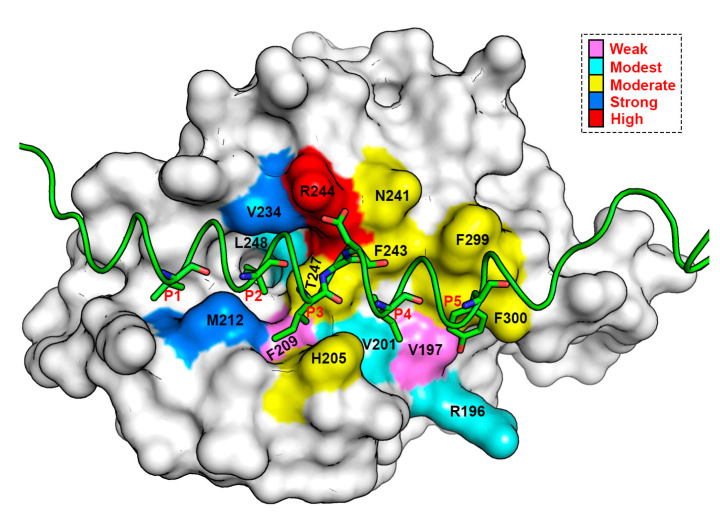
The residues present in the CBG (mMcl1—Bak complex) highlighted in distinctive segments with respect to the binding strength obtained from the PRD analysis.

Initially, an average value was calculated using the BFEs of the individual residues present at the CBG of mMcl1. Subsequently, the average values were used to quantify the binding pocket residues based on the binding strength. Figure 9 highlights the distinctive segments on the residues involved in the CBG of mMcl1 constructed based on the energy contributions. Among the residues involved at the CBG of mMcl1, R244 contributed the maximum energy consistent in all PAPs, i.e., its average energy value is as high as −5 kcal/mol. These higher energy contributions suggest that the residue present at this specific position plays a predominant role for the binding partner selectivity. Similarly, the average BFE values obtained for an ensemble of residues—H205, N241, G243, T247, F299 and F300—displayed moderate energy contributions, i.e., approximately −2 kcal/mol, respectively. 

This result shows that these residues play a significant role in complex formation. Furthermore, the average BFE values obtained for the group of residues—R196, V201 and L248—involved at the CBG exhibited modest energy values, i.e., somewhat more than −1 kcal/mol, respectively. This suggests that these residues are involved in additional support to the complex formation. Likewise, the average BFE values obtained for a couple of residues—V197 and F209—present at the CBG contributed only insufficient or weak energies, i.e., lower than –1 kcal/mol. This illustrates that these residues play a less important role in the complex formation. 

Overall, the predicted BFE values exhibited by the interface residues demonstrate that the higher energy contributing residues may together be responsible for engaging a selective PAP to the CBG of mMcl1. Moreover, the weak and insufficiently energy contributing residues also provided additional support for the PAPs to remain anchored in the CBG of mMcl1. 

### 3.7. Crucial Residues Involved in Allosteric Signal Transmission (AST) in mMcl1—PAPs Complexes

**Figure 10:**
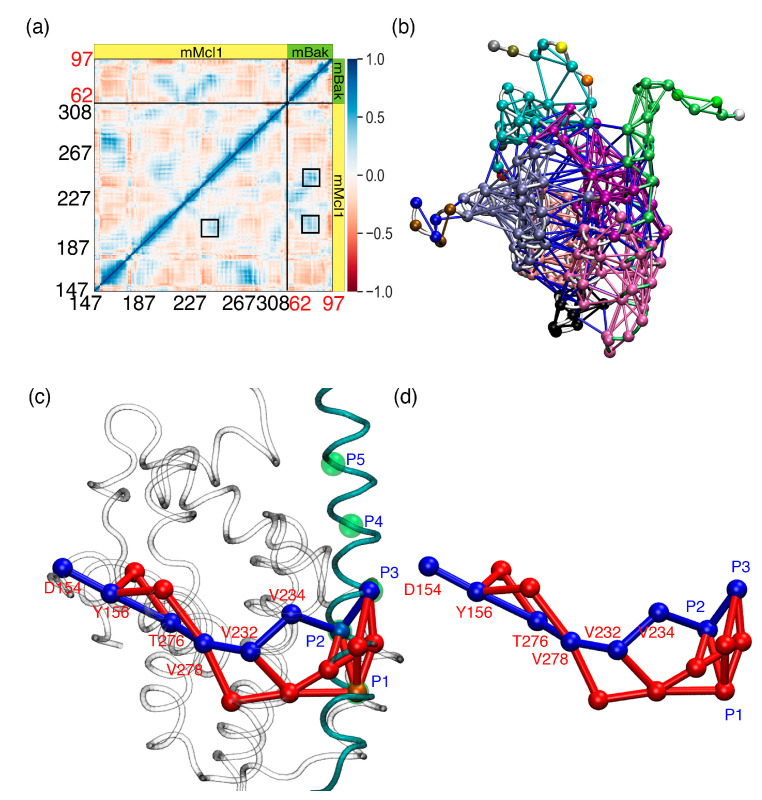
The allosteric signal transmission (AST) pathways predicted from the MD simulation (mMcl1—Bak complex selected as the representative). (**a**) The cross-correlation plot calculated for mMcl1—Bak complex. The correlation values range from –1 to +1 representing anti-correlated (red) and correlated (blue) regions. The critical residues that exhibit strong cross-correlations are highlighted in black boxes. (**b**) Dynamic network calculated for all residues in mMcl1—Bak complex based on coarse-grained community network analysis highlighting the presence of multiple communities in different colors. The network is built based on critical nodes identified based on unique identity numbers obtained from the NetworkView tool [55]. The nodes (spheres) communicate between each other through the connecting edges (lines) weighted based on betweeness centrality. (**c**) The predicted AST is overlaid on three-dimensional (3D) complex of mMcl1—Bak. The mMcl1 and Bak are represented in cartoon loop and highlighted in white and cyan colors, respectively. All critical nodes (spheres—highlighted in blue and red colors) represent the evidence for optimal and sub-optimal AST pathways between mMc1 and Bak, respectively. The green spheres highlighted in PAP represent the positions of five conserved hydrophobic residues (P1 to P5). (**d**) The AST path alone is displayed without mMcl1—Bak complex.

Despite, our MD simulations exhibited the favorable energy contributing residues at the CBG of mMcl1—PAP complexes, the mechanism of the collective internal motion of these residues involved AST remain unclear. This investigation might provide the molecular origin of the regulation, network of interaction across dimer interface, and putative allosteric path from one site to distal functional site [77]. For this, the advanced post-processing method was applied to MD trajectory (Figure 10). This method works based on (a) cross-correlations between residues and (b) coarse-grained community network analysis applied to all residues in a protein-peptide complex.

Despite Mcl1 plays a vital role in anti-apoptotic activity, not much is known about how the AST from one site (initiation) to the other site (distal) of the complex occurs. Here, the MD simulation corresponding to the mMcl1—PAP complex that exhibits a high binding affinity value (mMcl1—Bak complex) was selected as the representative. Subsequently, to gain more understanding on the potential AST, the dynamic network analysis was performed using the MD simulation. 

### 3.8. Cross-Correlation Analysis to Investigate Collective Internal Dynamics of mMcl1–Bak Complex

Figure 10a shows the cross-correlation results obtained from the MD simulations of mMcl1—Bak complex. The correlation map is useful to clearly distinguish the regions in the protein that show strong correlation and anti-correlation due to peptide binding. The diagonal region in the map shows strong correlation (blue color; +1) of each residue related to its own, while the distinct red spots highlighting the strong anti-correlated region (−1). Particularly, the residues at P2 (L75) and P3 (I78) region of the Bak peptide, and the V234 residue located at the CBG of mMcl1 shows strong correlated motion. Interestingly, these residues were pointed out as highly favorable energy contributing residues by our PRD analysis. Moreover, the residues involved in conserved polar interactions (between D80 from Bak and N241 and R244 of mMcl1, respectively; Figure 2d) also displays strong correlated motion. Regions corresponding to these residues were highlighted in boxes on the correlation map. Apart from these strong correlated regions, other residues from the off-diagonal regions also display strong correlations. These residues are the part of collective internal dynamics of the mMcl1—Bak complex. 

### 3.9. Community Network Analysis to Investigate Collective Internal Dynamics of mMcl1—Bak Complex

Although cross-correlation analysis highlights crucial residues that are involved in mMcl1—Bak complex stability, the analysis was extended to elucidate AST pathway. As network analysis can provide the evidence for allosteric communication between protein–peptide complex [78], the MD simulations of mMcl1—Bak complex was subjected to (i) all-residue dynamic network analysis and (ii) coarse-grained community network analysis (Figure 10b). Therefore, all residues in mMcl1—Bak complex were considered for network construction. Here, each amino acid residue was assigned as a node and the pair of nodes that are connected between each other within a certain distance through edges. Subsequently, the entire network was partitioned into clusters of communities (Figure 10c). The list of residues and the total number of the communities were given in the Table 5. The analysis produced 17 communities based on consensus correlation matrix. 

In order to obtain the long-range communication path, optimal and sub-optimal analysis was carried out. For this, the densely populated communities were alone considered (1, 10–17) while sparsely populated communities were ignored (2–9). The analysis produced ten sub-optimal pathways (Figure 10c,d). Subsequently, the pathway that connects two remote nodes spanning across the binding interface of mMcl1—Bak complex was alone selected. The selected optimal pathway identified the nodes (I78:P3→L75:P2 (Bak)→V234→V232→V278→T276→Y156→D154 (mMcl1)) from three different communities (13, 14 and 16; ref Table 5) demonstrating the long-range communication. This indicates that these residues are dynamically stable and have an important role in allosteric activity. Among the residues involved in the predicted pathway, L75, I78 from Bak and V234 from mMcl1 were located at the interface region. Moreover, these residues exhibited more favorable energy contributions from our PRD analysis. Interestingly, majority of the residues involved in the selected pathway are highly hydrophobic, except T276, Y156, and D154. 

It is important to mention that until now the Bcl2 family protein structure, particularly Mcl1, was investigated in the carboxy terminal region, while the amino terminal region was ignored due to its intrinsically unstructured nature, comprising the PEST (proline, glutamic acid, serine and threonine) sequence that targets proteasomal degradation, sites specific to ubiquitination, caspase cleavage and most importantly multiple phosphorylation [79]. As it is evident that the CBG of Mcl1 is located in the carboxy terminal region, where all the members of endogenous PAPs [22], synthetic peptides with BH3 mimetic [16], and non-peptide small molecules [80] bind to elicit subsequent cascade reaction, we took advantage to explore its thermodynamic properties. Based on this fact, it is plausible that the predicted energies from our investigation would vary when the full-length mMcl1 would be considered. Therefore, further experimental data would be needed to validate our findings.

Prior to our simulation, it was not clear which (i) thermodynamic components and (ii) high energy contributing residues are responsible for the mMcl1—PAP hetero-dimerization. Here, we have predicted crucial structural characteristics required for novel peptide or non-peptide organic small molecules synthesis. The results from the current investigation may efficiently provide valuable insight for the novel anti-apoptotic inhibitor design, particularly to down-regulate the activity of Mcl1. 

## 4. Conclusions

Before a novel peptide or organic chemical inhibitor synthesis, it is necessary to explore the binding mechanism of the protein molecule together with its existing binding partners. In the light of that, the present study attempts to comprehend the molecular basis of PAPs binding to mMcl1. A study conducted by Ku. B. et al., listed the diverse affinity values exhibited by different PAPs when binding with mMcl1 [22]. This information is used in the current investigation as the starting point to explore the molecular basis of different PAPs binding to mMcl1. In this process, initially the three-dimensional models of different mMcl1—PAP complexes were constructed based on the homology modeling approach. Subsequently, the MD–based MMGBSA approach was used to estimate the BFE values from the well-equilibrated phase for various PAPs binding to mMcl1 protein with three different GB models. The MD simulations revealed that the polar contacts, i.e., the salt bridge and the hydrogen bond formed between conserved aspartic acid of PAPs and the R244 and N241 residue of mMcl1, help to stabilize the complex in all the cases. Moreover, the other polar atoms found in the interface residues form the HB interactions to provide additional support to the mMcl1—PAP complexes. Furthermore, the BFE values obtained from the computational prediction methods (ΔG_mmgbsa_) showed excellent agreement with the experimental values (ΔG_expt_). Moreover, BFE analysis exhibited that the van der Waals (ΔG_vdw_) electrostatic (ΔG_ele_) and the gaseous (∆G_gas_) energies are highly favorable, while the polar (∆G_pol_) and solvation (∆G_sol_) energy component contributed unfavorable energies to the hetero-dimerization. Furthermore, the RIN helped to understand the interaction pattern for the highly conserved hydrophobic residues of PAPs. The RIN analysis visually clarified that the P2 position of the PAPs is surrounded with a maximum number of residues from the CBG of mMcl1, compared to the other positions of PAPs. This result is further supported by the decomposition analysis, which revealed that the leucine residue present at the P2 position of PAPs, and the F209, V234, and R244 residues present in the CBG of mMcl1 contribute higher energies in all the cases. Other studies focusing on mutational investigation at the P2 site of the PAPs revealed that binding to AAPs is significantly affected [74,75]. It is important to notice that V234 is one of the surrounding residues from mMcl1 to the P2 position of PAPs. Interestingly, the BFE values exhibited by the GD doublet were modest and also consistent across the complexes. The decomposition analysis also reveals that the presence of the charged residues at the P1 position of the PAPs reduced the binding affinity value by half. Finally, the results obtained from the cross-correlation analysis highlights the residues that are strong correlated motion, which are the part of collective internal dynamics. Furthermore, all-residue dynamic network analysis and coarse-grained community network-analysis produced clusters of communities based on consensus correlation matrix. Subsequently, the long-range optimal communication path was obtained from three different communities, indicates that these residues are dynamically stable. Overall, the identification of hot spot residues from this study is necessary for the interaction, and a better structural understanding of the PAPs binding modes will be crucial. In order to further substantiate our findings, additional experimental validations are required. The methods used in the current investigation may provide a better understanding towards mMcl1—PAP heterodimerization. Moreover, the current results may assist the designing process of novel PAP inhibitors, to down-regulate the Mcl1 activity.

## Figures and Tables

**Table 1:** The binding free energy values (BFEs—kcal/mol) calculated for different mMcl1—pro-apoptotic proteins (PAP) complexes using three different igb models (ref. method) during the last 10 ns of the molecular dynamics (MD) simulation. The experimental BFEs were obtained from the literature and converted to ∆G_bind_ using ΔG_bind_ = –RTln(K_D_) [22]. Here, the igb model developed by Tsui and case [37] performed better than the other two igb model used, and thus is used for the current study.

Peptides	K_D_ (nM)	ΔG_bind_	igb = 1	igb = 2	igb = 5
**mBak**	1.33	−12.05	−135.91 ± 0.39	−107.01 ± 0.35	−103.07 ± 0.37
**mBid**	1.55	−11.96	−132.66 ± 0.45	−105.79 ± 0.40	−99.81 ± 0.42
**mBim**	2.16	−11.77	−125.26 ± 0.34	−94.53 ± 0.29	−83.26 ± 0.32
**mPuma**	2.62	−11.65	−124.60 ± 0.31	−95.41 ± 0.28	−84.97 ± 0.31
**mNoxaB**	14	−10.67	−113.63 ± 0.59	−84.37 ± 0.51	−71.03 ± 0.54
**mNoxaA**	36.9	−10.09	−111.86 ± 0.46	−88.69 ± 0.39	−83.16 ± 0.40
**mBax**	39.5	−10.05	−109.12 ± 0.44	−82.81 ± 0.34	−71.39 ± 0.36
**mHrk**	44.8	−9.98	−106.54 ± 0.44	−80.12 ± 0.35	−67.36 ± 0.38
**mBmf**	446	−8.62	−103.73 ± 0.40	−74.39 ± 0.33	−62.46 ± 0.36
**mBik**	658	−8.39	−95.75 ± 0.33	−70.35 ± 0.30	−56.26 ± 0.32

**Table 2:** The individual components of the BFE (kcal/mol) values obtained from igb = 1 for different mMcl1—PAP complexes during the last 10 ns of the MD simulation.

PAP/BFE	ΔG_vdw_	ΔG_ele_	ΔG_pol_	ΔG_non−pol_	ΔG_sol_	ΔG_gas_	ΔG_mmgbsa_	ΔG_bind_	K_D_ (nM)
**mBak**	−132.86 ± 0.29	−233.23 ± 1.67	249.02 ± 1.49	−18.83 ± 0.03	230.18 ± 1.48	−366.10 ± 1.71	−135.91 ± 0.39	−12.05	1.33
**mBid**	−140.93 ± 0.44	−294.83 ± 2.30	331.54 ± 2.09	−18.85 ± 0.05	369.33 ± 2.35	−501.99 ± 2.69	−132.66 ± 0.45	−11.96	1.55
**mBim**	−133.19 ± 0.30	−389.53 ± 2.17	415.97 ± 2.02	−18.51 ± 0.03	397.46 ± 2.01	−522.73 ± 2.18	−125.26 ± 0.34	−11.77	2.16
**mPuma**	−132.88 ± 0.24	−391.12 ± 1.42	417.42 ± 1.27	−18.02 ± 0.03	399.40 ± 1.25	−524.01 ± 1.45	−124.60 ± 0.31	−11.65	2.62
**mNoxaB**	−127.22 ± 0.42	−142.90 ± 1.94	173.58 ± 1.77	−17.09 ± 0.06	156.49 ± 1.74	−270.13 ± 2.11	−113.63 ± 0.59	−10.67	14.0
**mNoxaA**	−123.29 ± 0.40	−148.49 ± 2.12	177.21 ± 2.12	−17.29 ± 0.05	159.92 ± 2.09	−271.78 ± 2.29	−111.86 ± 0.46	−10.09	36.9
**mBax**	−135.79 ± 0.30	−62.54 ± 2.25	106.89 ± 2.11	−17.67 ± 0.04	70.17 ± 1.97	−181.28 ± 2.10	−109.12 ± 0.44	−10.05	39.5
**mHrk**	−120.76 ± 0.28	−110.34 ± 3.28	140.73 ± 3.07	−16.17 ± 0.04	124.56 ± 3.04	−231.11 ± 3.37	−106.54 ± 0.44	−9.98	44.8
**mBmf**	−129.10 ± 0.33	−6.00 ± 1.57	48.56 ± 1.40	−17.19 ± 0.05	31.37 ± 1.39	−135.11 ± 1.61	−103.73 ± 0.40	−8.62	446
**mBik**	−116.65 ± 0.33	−119.83 ± 1.52	156.12 ± 1.35	−15.39 ± 0.04	140.72 ± 1.35	−236.48 ± 1.46	−95.75 ± 0.33	−8.39	658

ΔG_vdw_—van der Waals interactions; ΔG_ele_—Electrostatic interactions; ΔG_pol_—Polar interactions; ΔG_non−pol_—Non-polar interactions; ΔG_sol_—Solvation of BFEs; ΔG_gas_—Molecular mechanics of BFEs; ΔG_mmgbsa_—Molecular Mechanics—Generalized Born Solvent Accessibility; ΔG_bind_—Binding free energies converted from experimental values using ΔG_bind_ = –RTln(K_D_); K_D_ (nM)—Binding constant from experimental values.

**Table 3:** The per-residue decomposition (PRD—kcal/mol) analysis for the conserved residues of PAPs interacting with mMcl1.

PAP/Pockets	Five Conserved Hydrophobic Residue Positions						
	P1	P2	P3	G/A	D	P4	P5
**mBak**	−4.53 ± 0.02	−6.78 ± 0.02	−3.91 ± 0.02	−1.57 ± 0.03	−2.47 ± 0.01	−3.88 ± 0.02	−2.86 ± 0.02
**mBid**	−4.87 ± 0.02	−6.54 ± 0.02	−3.76 ± 0.02	−1.65 ± 0.04	−2.67 ± 0.01	−3.75 ± 0.02	−3.98 ± 0.02
**mBim**	−5.65 ± 0.02	−6.12 ± 0.02	−3.71 ± 0.02	−1.45 ± 0.03	−2.09 ± 0.01	−5.18 ± 0.02	−3.63 ± 0.03
**mPuma**	−4.29 ± 0.02	−6.67 ± 0.02	−4.31 ± 0.02	−1.66 ± 0.02	−3.08 ± 0.01	−5.24 ± 0.02	−2.46 ± 0.02
**mNoxaB**	−0.29 ± 0.04	−6.85 ± 0.03	−3.33 ± 0.02	−2.65 ± 0.04	−2.73 ± 0.02	−3.79 ± 0.02	−4.50 ± 0.04
**mNoxaA**	−6.19 ± 0.03	−6.43 ± 0.02	−4.53 ± 0.03	−0.11 ± 0.01	−1.92 ± 0.01	−4.47 ± 0.02	−5.89 ± 0.06
**mBax**	−4.94 ± 0.03	−5.92 ± 0.02	−3.98 ± 0.02	−1.36 ± 0.03	−2.06 ± 0.01	−4.36 ± 0.02	−3.49 ± 0.02
**mHrk**	−2.90 ± 0.02	−5.97 ± 0.02	−4.08 ± 0.02	−1.10 ± 0.02	−2.14 ± 0.01	−5.17 ± 0.02	−1.71 ± 0.01
**mBmf**	−4.80 ± 0.03	−5.81 ± 0.02	−4.22 ± 0.02	−0.34 ± 0.00	−2.33 ± 0.01	−5.72 ± 0.03	−2.84 ± 0.02
**mBik**	−4.26 ± 0.02	−6.67 ± 0.02	−3.52 ± 0.02	−1.28 ± 0.02	−2.43 ± 0.01	−4.14 ± 0.02	−3.33 ± 0.02

**Table 4:** The PRD (kcal/mol) analysis for mMcl1 residues interacting with PAPs.

PAPs/Residues	Residues Present in the Canonical Binding Groove of mMcl1						
	R196	V197	V201	H205	F209	M212	V234
**mBak**	0.10 ± 0.00	−0.57 ± 0.01	−1.05 ± 0.13	−2.51 ± 0.31	−0.97 ± 0.12	−4.00 ± 0.25	−2.54 ± 0.13
**mBid**	−6.63 ± 0.05	−0.54 ± 0.00	−1.96 ± 0.14	−2.63 ± 0.29	−0.79 ± 0.09	−3.82 ± 0.21	−2.35 ± 0.21
**mBim**	−0.19 ± 0.00	−0.91 ± 0.01	−2.20 ± 0.18	−1.52 ± 0.31	−0.79 ± 0.09	−3.67 ± 0.21	−3.04 ± 0.28
**mPuma**	−4.53 ± 0.10	−1.53 ± 0.01	−1.48 ± 0.16	−3.00 ± 0.30	−0.83 ± 0.08	−3.09 ± 0.21	−3.81 ± 0.19
**mNoxaB**	0.07 ± 0.00	−0.36 ± 0.00	−0.80 ± 0.08	−1.34 ± 0.15	−0.92 ± 0.08	−3.66 ± 0.22	−3.48 ± 0.21
**mNoxaA**	−3.47 ± 0.15	−1.57 ± 0.01	−2.08 ± 0.22	−2.10 ± 0.29	−0.85 ± 0.07	−3.73 ± 0.21	−3.36 ± 0.29
**mBax**	−2.07 ± 0.02	−2.13 ± 0.01	−1.03 ± 0.13	−1.69 ± 0.45	−0.83 ± 0.09	−3.15 ± 0.23	−4.58 ± 0.23
**mHrk**	0.21 ± 0.00	−0.19 ± 0.00	−0.94 ± 0.09	−1.62 ± 0.30	−0.93 ± 0.08	−3.88 ± 0.26	−3.71 ± 0.20
**mBmf**	−0.10 ± 0.00	−1.24 ± 0.02	−2.54 ± 0.21	−3.14 ± 0.41	−0.75 ± 0.09	−3.60 ± 0.23	−3.50 ± 0.30
**mBik**	0.10 ± 0.00	−0.25 ± 0.00	−0.85 ± 0.11	−1.32 ± 0.27	−0.98 ± 0.12	−4.35 ± 0.22	−2.55 ± 0.16
	**N241**	**G243**	**R244**	**T247**	**L248**	**F299**	**F300**
**mBak**	−2.01 ± 0.41	−2.93 ± 0.3	−4.99 ± 0.76	−1.88 ± 0.18	−1.44 ± 0.13	−1.05 ± 0.37	−1.44 ± 0.18
**mBid**	−3.77 ± 0.34	−3.66 ± 0.29	−6.68 ± 0.69	−2.28 ± 0.18	−0.82 ± 0.09	−1.52 ± 0.22	−1.96 ± 0.22
**mBim**	−1.36 ± 0.19	−2.08 ± 0.24	−4.86 ± 0.72	−2.43 ± 0.20	−1.05 ± 0.09	−2.85 ± 0.36	−3.42 ± 0.34
**mPuma**	−1.22 ± 0.15	−1.73 ± 0.16	−4.88 ± 0.60	−2.44 ± 0.15	−1.07 ± 0.11	−3.38 ± 0.26	−2.86 ± 0.31
**mNoxaB**	−3.36 ± 0.32	−2.68 ± 0.24	−8.18 ± 0.89	−2.80 ± 0.23	−0.67 ± 0.08	−3.93 ± 0.42	−2.74 ± 0.27
**mNoxaA**	−0.82 ± 0.23	−2.07 ± 0.23	−2.69 ± 0.37	−2.43 ± 0.23	−1.19 ± 0.10	−1.00 ± 0.22	−1.79 ± 0.31
**mBax**	−3.06 ± 0.84	−3.19 ± 0.46	−5.71 ± 0.83	−1.89 ± 0.22	−1.16 ± 0.12	−0.17 ± 0.07	−1.07 ± 0.20
**mHrk**	−1.30 ± 0.16	−2.61 ± 0.24	−4.64 ± 0.54	−2.19 ± 0.21	−0.93 ± 0.10	−3.35 ± 0.51	−2.40 ± 0.25
**mBmf**	−0.06 ± 0.04	−1.45 ± 0.16	−3.09 ± 0.53	−2.33 ± 0.17	−0.98 ± 0.10	−2.22 ± 0.32	−1.16 ± 0.14
**mBik**	−1.66 ± 0.30	−3.07 ± 0.30	−5.44 ± 0.61	−1.95 ± 0.23	−0.86 ± 0.09	−4.11 ± 0.35	−1.24 ± 0.19

**Table 5:** List of communities and its members.

Communities	Residues Involved
1	147, 149, 151
2	175
3	177
4	302
5	304
6	307
7	308
8	93, 95
9	78
10	148, 150, 152
11	165–173, 253, 255–269, 271
12	174, 176, 178–193, 195, 293–294, 297, 299, 301, 303, 305–306
13	194, 196–206, 208–209, 237, 239, 241–252, 254,2 91, 295–296, 298, 300, 78–80, 82
14	75, 77, 81, 83–92, 94, 96
15	207, 210–216, 226, 228, 229–236, 238
16	153–164, 240, 270, 272–290, 292
17	217–225, 227

## Supplementary Materials

The following are available online at https://www.mdpi.com/2218-273X/10/8/1114/s1, Figure S1: (a) The conserved residues of PAPs (left—helix and right—molecular surface) form the hydrophobic face (sticks) that potentially interact to the binding groove of anti-apoptotic mMcl1, Figure S2: Closer views of the hydrophobic face of (stick of) PAPs [(a) Bid, (b) Bim, (c) Puma, (d) NoxaB, (e) NoxaA, (f) Bax (g) Hrk (h) Bmf, and (i) Bik] interacting at the sub-pockets (P1-P5) present inside the binding groove of mMcl1 (molecular surface representation), Figure S3: (a) The root-mean-squared deviation (*rmsd*) values calculated using Cα atoms for each mMcl1—PAP complex relative to its initial coordinates over the period of time. (b) The violin plots were constructed using the trajectories from last 10 ns. The plot displays the distribution and mean (black dot) *rmsd* values for each mMcl1—PAP complex, Figure S4: The polar contacts (black dotted lines) identified between the anti-apoptotic mMcl1 (white) and PAPs [(a) Bid: D95—cyan, (b) Bim: D155—salmon, (c) Puma: D146—purple, (d) NoxaB: D83—orange, (e) NoxaA: D32—yellow (f) Bax: D68—marine blue (g) Hrk: D42—light green (h) Bmf: D229—light blue, and (i) Bik: D60—pink] obtained using an average snapshot collected from the equilibrated phase of MD simulation, Figure S5: Total number of polar contacts at mMcl1—PAP interface over the time period (ns). Table S1: The polar atom distances (Å) between mMcl1 and PAPs measured using an average snapshot collected from the equilibrated phase of MD simulation. The distances were calculated using our in-house program *surf* (Johnson, MS—Unpublished). The distance cut-off was set to 3.2 Å. The ’*’ sign represents the polar atoms from different BH3 peptides.

## Abbreviations

Bcl2	B-cell lymphoma 2
Mcl1	Myeloid cell leukemia
BH3	Bcl-2 homology
AAP	Anti-apoptotic proteins
PAP	Pro-apoptotic proteins
BFE	Binding free energy
PRD	Per–residue decomposition
