# Supplementary material for: Predicted Hotspot Residues Involved in Allosteric Signal Transmission in Pro-Apoptotic Peptide—Mcl1 Complexes
Source: Biomolecules. 2020 Jul 28;10(8):1114. doi: 10.3390/biom10081114 (PMC7463671; doi:10.3390/biom10081114)
Supplement: Supplementary file 1 [file biomolecules-10-01114-s001.pdf]

Supplementary Material

Predicted hotspot residues involved in allosteric signal transmission in Pro-apoptotic Peptide—Mcl1 Complexes

Parthiban Marimuthu[1†], Jamoliddin Razzokov[2], Kalaimathy Singaravelu[3], and Annemie Bogaerts[2]

[1]Structural Bioinformatics Laboratory (SBL), Biochemistry and Pharmacy, Faculty of Science and Engineering, Åbo Akademi University, FI-20520 Turku, Finland.

[2]PLASMANT Research Group, Chemistry Department, University of Antwerp, 2610 Antwerp, Belgium

[3]Department of Future Technologies, Faculty of Science and Engineering, University of Turku, FI-20014, Turku, Finland.

[†]Address correspondence to:

Dr. Parthiban Marimuthu (Ph.D.), Structural Bioinformatics Laboratory (SBL), Pharmacy, Faculty of Science and Engineering, Åbo Akademi University, Tykistökatu 6A, FI-20520 Turku, Finland. Phone: +358 2 215 4600, E-mail address: parthiban.marimuthu@abo.fi

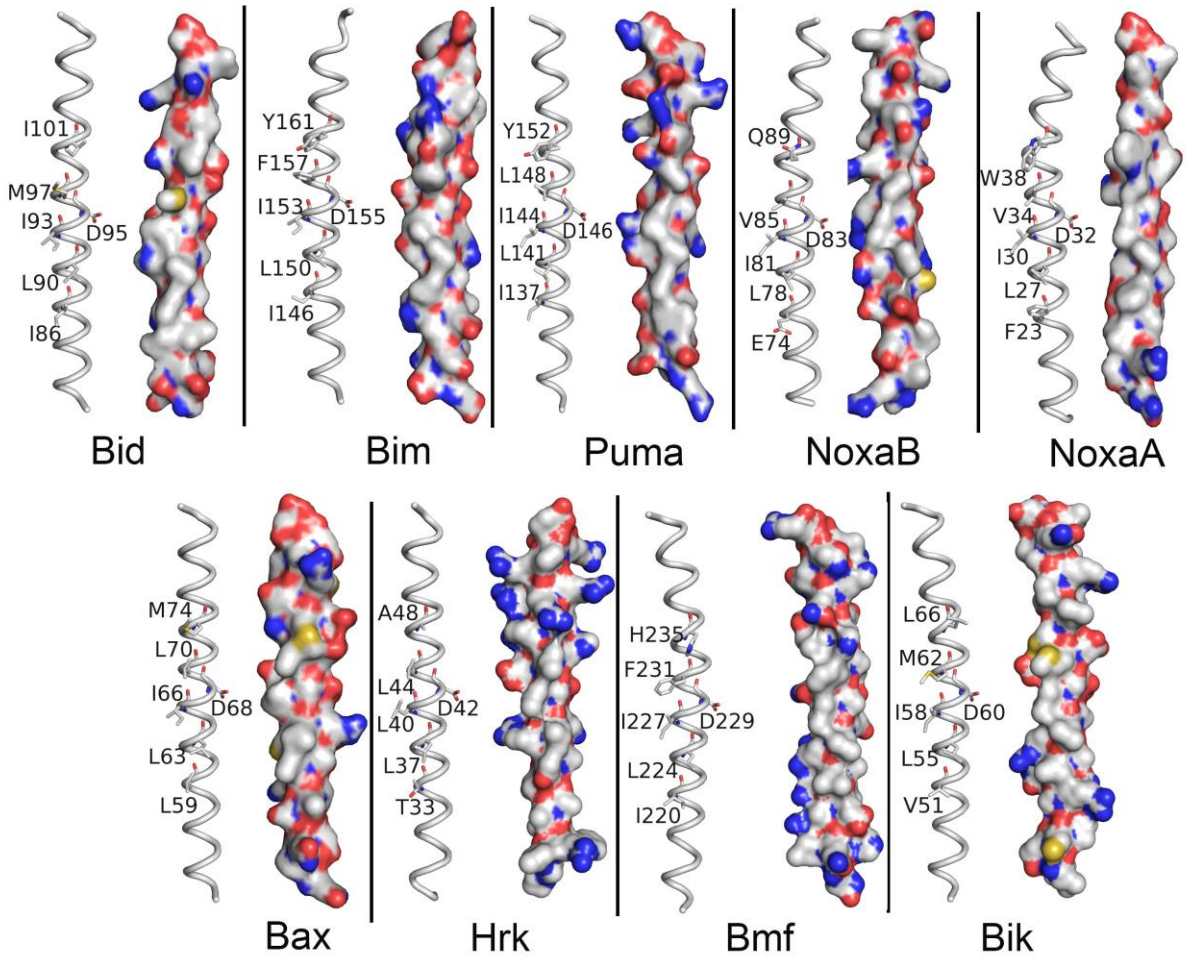

Figure S1. (a) The conserved residues of PAPs (left — helix and right —molecular surface) form the hydrophobic face (sticks) that potentially interact to the binding groove of anti-apoptotic mMcl1.

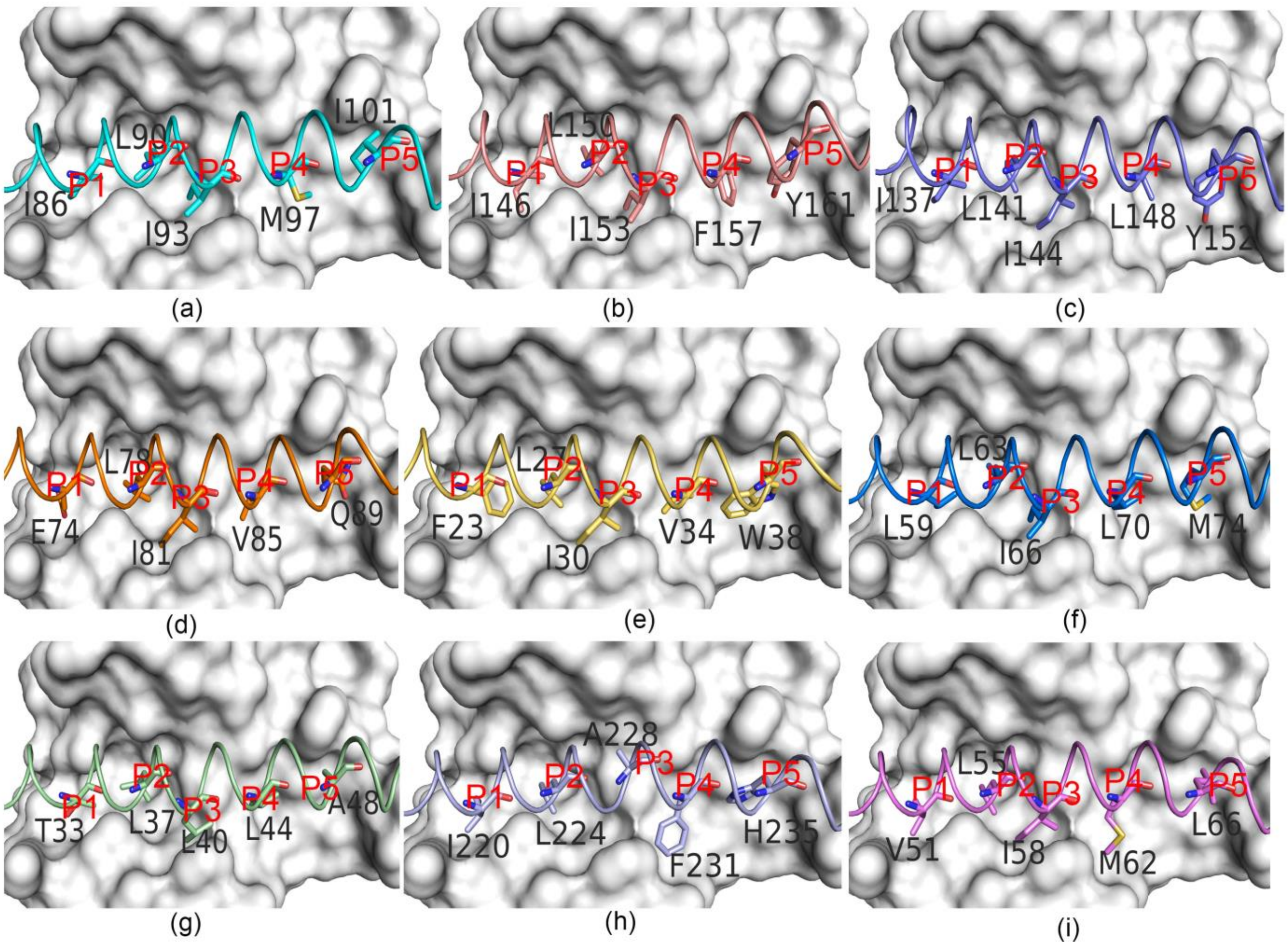

Figure S2: Closer views of the hydrophobic face of (stick of) PAPs [(a) Bid, (b) Bim, (c) Puma, (d) NoxaB, (e) NoxaA, (f) Bax (g) Hrk (h) Bmf, and (i) Bik] interacting at the sub-pockets (P1-P5) present inside the binding groove of mMcl1 (molecular surface representation).

(a)

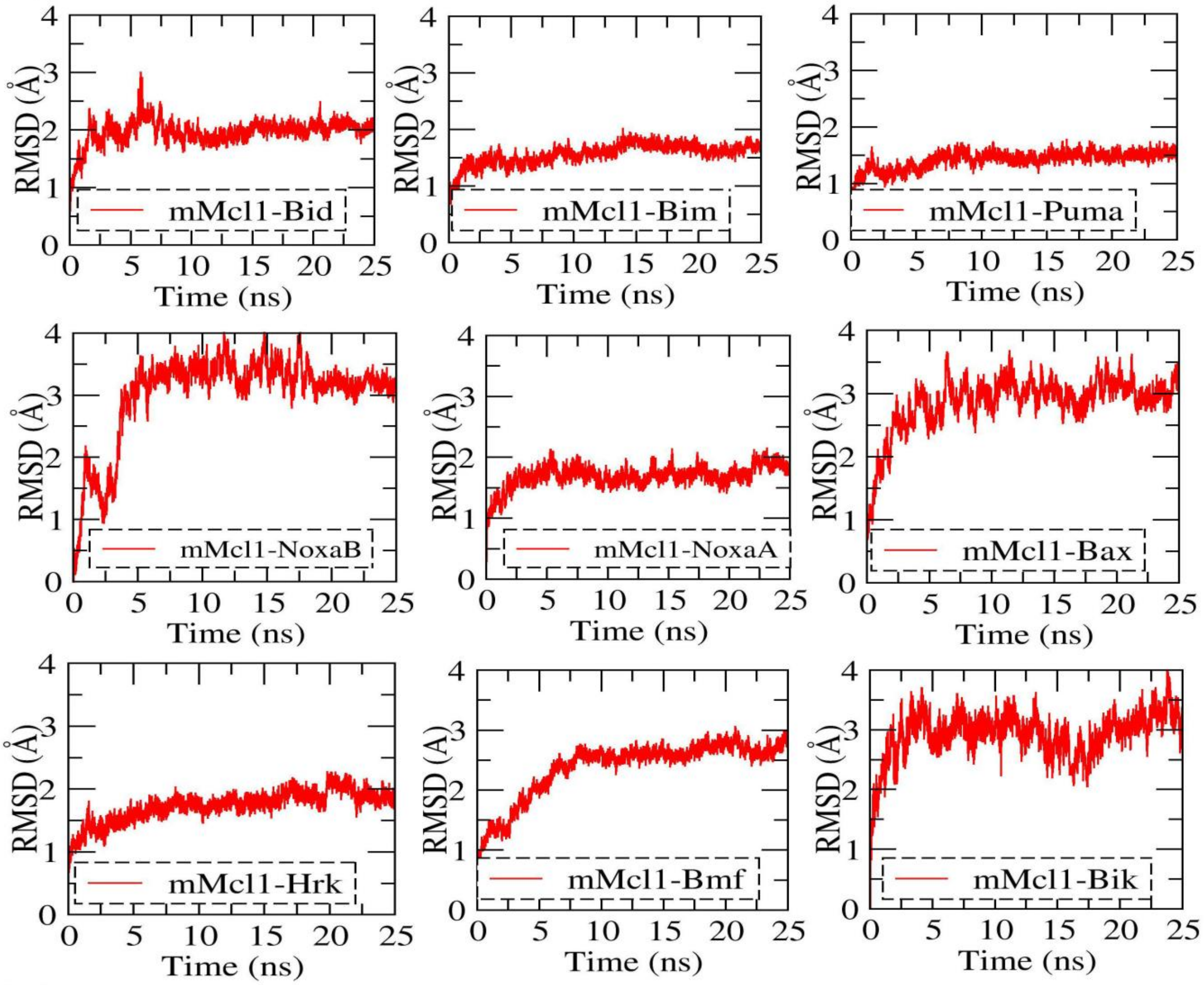

(b)

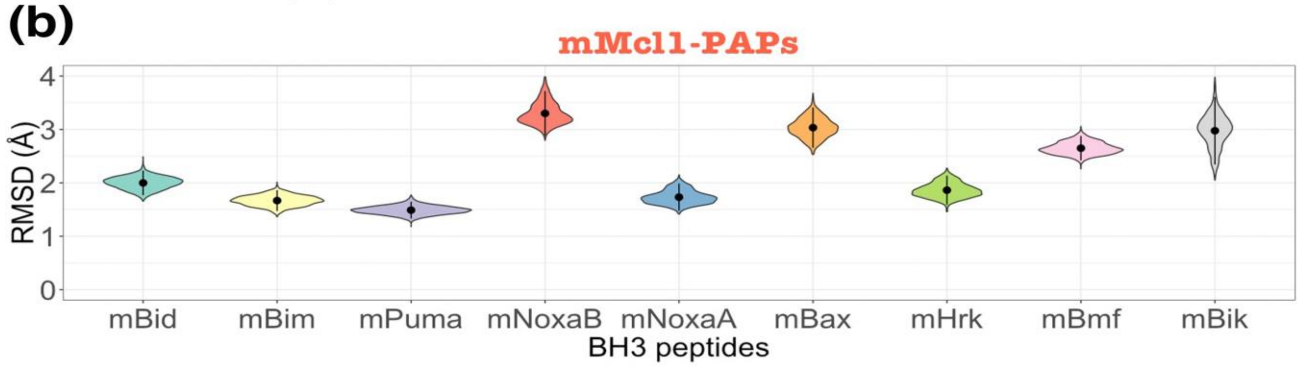

Figure S3: (a) The root-mean-squared deviation (*rmsd*) values calculated using Cα atoms for each mMcl1—PAP complex relative to its initial coordinates over the period of time. (b) The violin plots were constructed using the trajectories from last 10 ns. The plot displays the distribution and mean (black dot) *rmsd* values for each mMcl1—PAP complex.

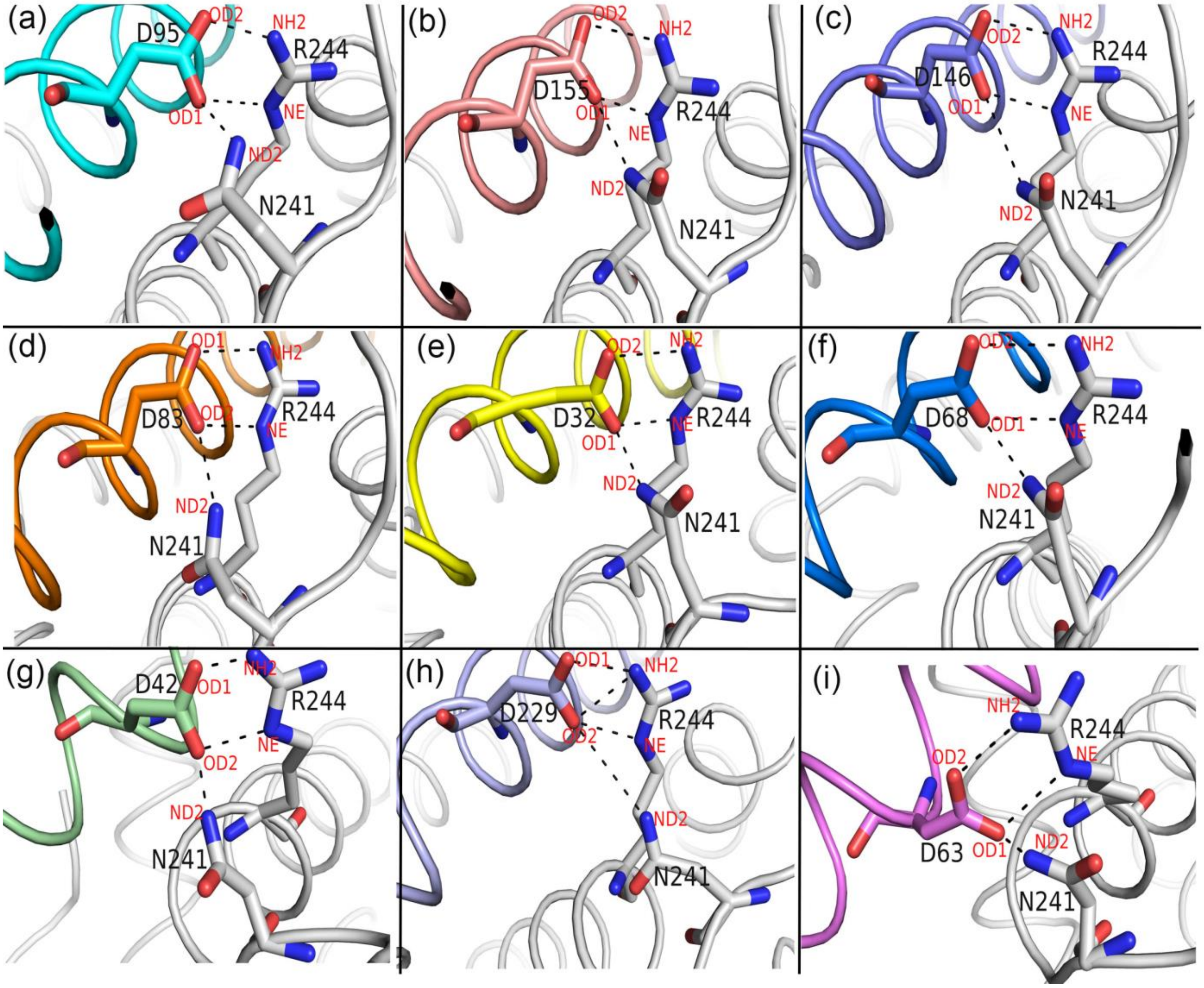

Figure S4: The polar contacts (black dotted lines) identified between the anti-apoptotic mMcl1 (white) and PAPs [(a) Bid: D95 - cyan, (b) Bim: D155 - salmon, (c) Puma: D146 - purple, (d) NoxaB: D83 - orange, (e) NoxaA: D32 – yellow (f) Bax: D68 - marine blue (g) Hrk: D42 – light green (h) Bmf: D229 – light blue, and (i) Bik: D60 - pink] obtained using an average snapshot collected from the equilibrated phase of MD simulation.

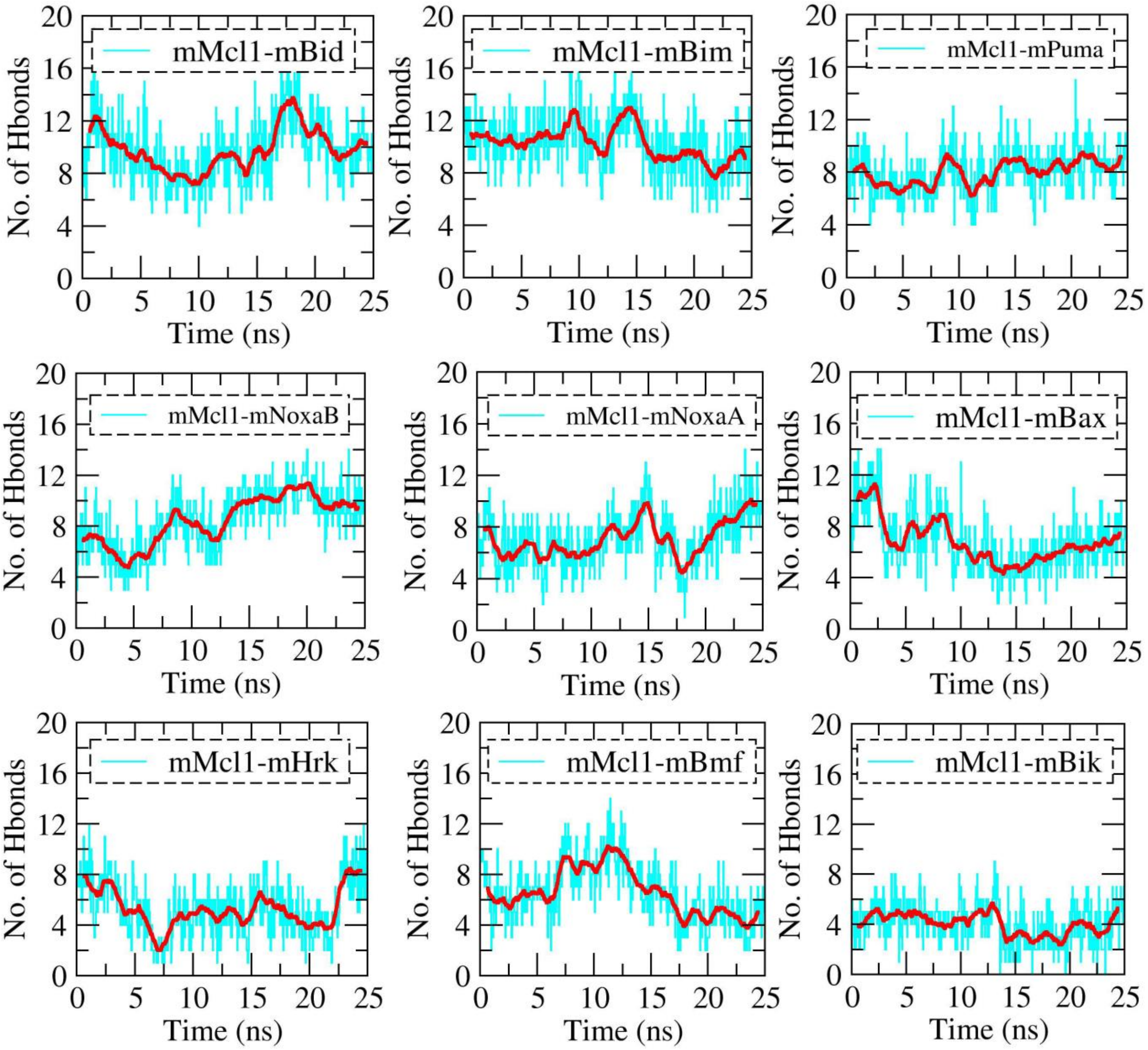

Figure S5: Total number of polar contacts at mMcl1—PAP interface over the time period (ns).

Table S1: The polar atom distances (Å) between mMcl1 and PAPs measured using an average snapshot collected from the equilibrated phase of MD simulation. The distances were calculated using our in-house program *surf* (Johnson, MS — Unpublished). The distance cut-off was set to 3.2 Å. The '*' sign represents the polar atoms from different BH3 peptides.

Peptides	Chain	Residue	Atom	Chain	Residue	Atom	Distance (Å)
mBak	A	K215	NZ	B*	Q74	OE1	2.7
		N241	ND2		D80	OD2	3.1
		G243	N		N83	OD1	3.2
		R244	NH2		D80	OD1	2.8
		R244	NH1		D80	OD2	2.9
		F299	O		Y86	OH	2.7
mBid	A	K215	NZ	B*	N85	OD1	2.9
		R229	NH1		E80	OE1	2.9
		R229	NH1		E80	OE2	2.9
		R229	NH2		E80	OE2	3.0
		H233	ND1		H84	NE2	2.9
		N241	ND2		D95	OD1	2.8
		G243	N		D98	OD1	3.0
		R244	NH1		D95	OD2	3.1
		R244	NH2		D95	OD1	3.0
mBim	A	R229	NH1	B*	E143	OE1	2.7
		R229	NH1		E143	OE2	3.1
		H233	O		R151	NH1	2.9
		N241	ND2		D155	OD1	3.0
		R244	NH2		D155	OD1	2.9
		R244	NH1		D155	OD2	2.9
mPuma	A	M156	N	B*	F299	O	2.8
		H205	NE2		I144	O	3.2
		R229	NE		E130	OE2	3.0
		R229	NH2		E130	OE2	2.7
		R229	NH2		E131	OE1	2.7
		H233	ND1		R142	NH1	3.0
		H233	O		R142	NH1	3.1
		R244	NH2		D146	OD1	2.8
		R244	NH1		D146	OD2	2.8
mNoxaB	A*	T65	OG1	B	K215	O	2.8
		K72	NZ		R229	O	3.2
		E74	OE1		K215	NZ	2.7
		L78	O		T247	OG1	2.9
		R79	NH1		H233	O	2.7
		D83	OD2		N241	ND2	2.8
		D83	OD1		R244	NH2	2.7

		D83	OD2		R244	NE	2.8
		Q89	NE2		F299	O	3.2
		Q89	NE2		F300	O	3.2
		Q89	NE2		V302	O	3.1
mNoxaA	A	K215	NZ	B*	E22	OE1	3.1
		K215	NZ		E22	OE2	3.2
		R229	NH2		E18	OE2	2.8
		N241	ND2		D32	OD2	3.1
		R244	NH1		D32	OD2	3.0
		R244	NH2		D32	OD1	2.9
mBax	A*	Q49	OE1	B	R229	NH1	2.8
		Q52	NE2		R229	O	2.9
		S60	OG		H233	ND1	3.1
		R64	NH1		H233	O	2.9
		R64	O		R244	NE	3.1
		D71	OD1		G243	N	2.9
mHrk	A	M212	O	B*	T33	OG1	3.2
		N241	ND2		H45	ND1	3.1
		N241	ND2		D42	OD2	2.1
		G243	N		H45	ND1	3.2
		R244	NH1		D42	OD2	2.8
		R244	NH2		D42	OD1	2.8
		V302	O		A53	N	2.8
mBmf	A	H205	NE2	B*	I227	O	2.8
		R229	O		H214	NE2	3.1
		V234	O		Q225	OE1	3.0
		R244	NH1		Q225	OE1	3.1
mBik	A*	L41	O	B	R229	NH1	2.7
		M42	O		R229	NH2	2.9
		Q50	OE1		K215	NZ	3.1
		D60	OD1		R244	OD1	3.2
		D60	OD2		R244	OD2	3.1
		R70	NH2		F299	O	2.9
